# Accuracy of stroke volume measurement with phase-contrast cardiovascular magnetic resonance in patients with aortic stenosis

**DOI:** 10.1186/s12968-021-00814-4

**Published:** 2021-11-04

**Authors:** Ezequiel Guzzetti, Hugo-Pierre Racine, Lionel Tastet, Mylène Shen, Eric Larose, Marie-Annick Clavel, Philippe Pibarot, Jonathan Beaudoin

**Affiliations:** Institut Universitaire de Cardiologie et de Pneumologie de Québec, Université Laval, 2725 Chemin Sainte-Foy, Québec, QC G1V-4G5 Canada

**Keywords:** Phase contrast, Aortic stenosis, Valvular heart disease

## Abstract

**Background:**

Phase contrast (PC) cardiovascular magnetic resonance (CMR) in the ascending aorta (AAo) is widely used to calculate left ventricular (LV) stroke volume (SV). The accuracy of PC CMR may be altered by turbulent flow. Measurement of SV at another site is suggested in the presence of aortic stenosis, but very few data validates the accuracy or inaccuracy of PC in that setting. Our objective is to compare flow measurements obtained in the AAo and LV outflow tract (LVOT) in patients with aortic stenosis.

**Methods:**

Retrospective analysis of patients with aortic stenosis who had CMR and echocardiography. Patients with mitral regurgitation were excluded. PC in the AAo and LVOT were acquired to derive SV. LV SV from end-systolic and end-diastolic tracings was used as the reference measure. A difference ≥ 10% between the volumetric method and PC derived SVs was considered discordant. Metrics of turbulence and jet eccentricity were assessed to explore the predictors of discordant measurements.

**Results:**

We included 88 patients, 41% with bicuspid aortic valve. LVOT SV was concordant with the volumetric method in 79 (90%) patients vs 52 (59%) patients for AAo SV (p = 0.015). In multivariate analysis, aortic stenosis flow jet angle was a strong predictor of discordant measurement in the AAo (p = 0.003). Mathematical correction for the jet angle improved the concordance from 59 to 91%. Concordance was comparable in patients with bicuspid and trileaflet valves (57% and 62% concordance respectively; p = 0.11). Accuracy of SV measured in the LVOT was not influenced by jet eccentricity. For aortic regurgitation quantification, PC in the AAo had better correlation to volumetric assessments than LVOT PC.

**Conclusion:**

LVOT PC SV in patients with aortic stenosis and eccentric jet might be more accurate compared to the AAo SV. Mathematical correction for the jet angle in the AAo might be another alternative to improve accuracy.

**Supplementary Information:**

The online version contains supplementary material available at 10.1186/s12968-021-00814-4.

## Background

Phase-contrast (PC) is the main approach to quantify blood flow parameters with cardiovascular magnetic resonance (CMR). It is widely accepted and used to assess valvular and congenital heart diseases [1, 2]. Flow volume can be measured by the acquisition of a cross-sectional image of the vessel or area of interest, in which fluid velocity is calculated for every pixel [1, 3]. However, the accuracy of PC may be altered by the presence of turbulent flow. Such turbulence typically occurs in patients with heart valve diseases and stenotic or regurgitant jets, which are associated with flow acceleration/deceleration and intravoxel dephasing [4–6]. Regurgitant or stenotic valves are also associated with flow eccentricity, increasing the difficulty to optimize the correct imaging plane.

PC in the ascending aorta (AAo) is widely used to calculate forward stroke volume (SV) and regurgitant aortic flow; those variables are essential to assess mitral and/or aortic regurgitation [5, 7]. Aortic regurgitation can be measured directly from the PC sequence, while mitral regurgitation is computed as the difference between aortic SV and left ventricular (LV) SV obtained volumetrically from a short axis stack. Blood flow quantification in the AAo is however potentially altered in the presence of aortic valve disease, and SV measurement at another site [pulmonary valve or left ventricular outflow tract (LVOT)] is sometime suggested [5, 8, 9]. However, it is not clear if and at which severity the presence of aortic valve disease can invalidate AAo PC measurements [10, 11], and there are few data comparing SV obtained from different sites in the presence of valve disease. These issues are relevant as patients with multiple valve diseases are frequently encountered and increasingly assessed by CMR [5, 7].

Our objectives are to compare flow measurements obtained by PC in the ascending aorta (SV_AAo_) and the LVOT (SV_LVOT_) in patients with various degrees of aortic stenosis. We have selected a population without significant mitral regurgitation so that SV obtained by volumetric method (SV_VM_) from LV tracings can be used as a reference.

## Methods

### Patient population

A total of 88 patients prospectively recruited in the ongoing PROGRESSA study (NCT 01679431) between 2011 and 2015 were retrospectively analyzed. Included patients had either aortic stenosis (Vmax > 2 m/s), bicuspid aortic valve (with or without stenosis), and controls without valve disease. Patients were excluded if they had symptomatic aortic stenosis, any mitral valve disease (mitral stenosis or > trace mitral regurgitation), LV ejection fraction (LVEF) < 50%, rheumatic valve disease or endocarditis, previous aortic/mitral valve repair or replacement, previous ascending aorta repair or replacement, if they were pregnant/lactating or if they had contraindications to gadolinium. More details about inclusion/exclusion criteria were previously described [12]. Patients underwent transthoracic echocardiogram (TTE) and CMR within 3 months. The study was approved by the Ethics Committee of the Quebec Heart and Lung Institute and patients signed a written informed consent at the time of inclusion.

### Doppler echocardiographic measurements

All Doppler echocardiographic examinations were acquired using commercially available ultrasound machines (iE33 and EPIQ, Philips Healthcare, Best, Netherlands) and according to the current recommendations of the American Society of Echocardiography [13, 14]. Images were analyzed offline in a core laboratory. Aortic regurgitation and mitral regurgitation were graded using a multiparametric approach as suggested by guidelines [14, 15]. All patients with more than mild mitral regurgitation severity were excluded for the purpose of this study.

### Cardiovascular magnetic resonance measurements

CMR was performed using 1.5 and 3T CMR scanners (Achieva or Ingenia, Philips Healthcare). Cardiac morphology and function were assessed by balanced steady-state free precession sequences at 30 phases per cardiac cycle in held end-expiration. Standard planes included 8–14 contiguous parallel short-axis (8 mm thickness, 0 mm gap) covering the entire cardiac volume, 2-chamber, 4-chamber and two orthogonal LVOT planes. Typical parameters at 1.5T were TR/TE 3.2/1.6 ms, flip angle 60º, and NEX of 1, in-plane spatial resolution of 1.6 × 2 mm. Equivalent acquisition parameters at 3T were TR/TE 2.8/1.3 ms, flip angle 45°, and NEX of 1, in-plane spatial resolution of 1.7 mm × 2 mm, 7 mm slice thickness, 0 mm gap. LV volumes and LVEF were measured by contour analysis of end-diastolic and end-systolic phases of the short-axis stack. LV SV_VM_ was calculated as the difference between LV end-diastolic and end-systolic volumes. To reflect different practices of CMR post-processing, LV volumes and SV were computed with and without including the papillary muscles and major trabeculations in the blood pool.

**Fig. 1:**
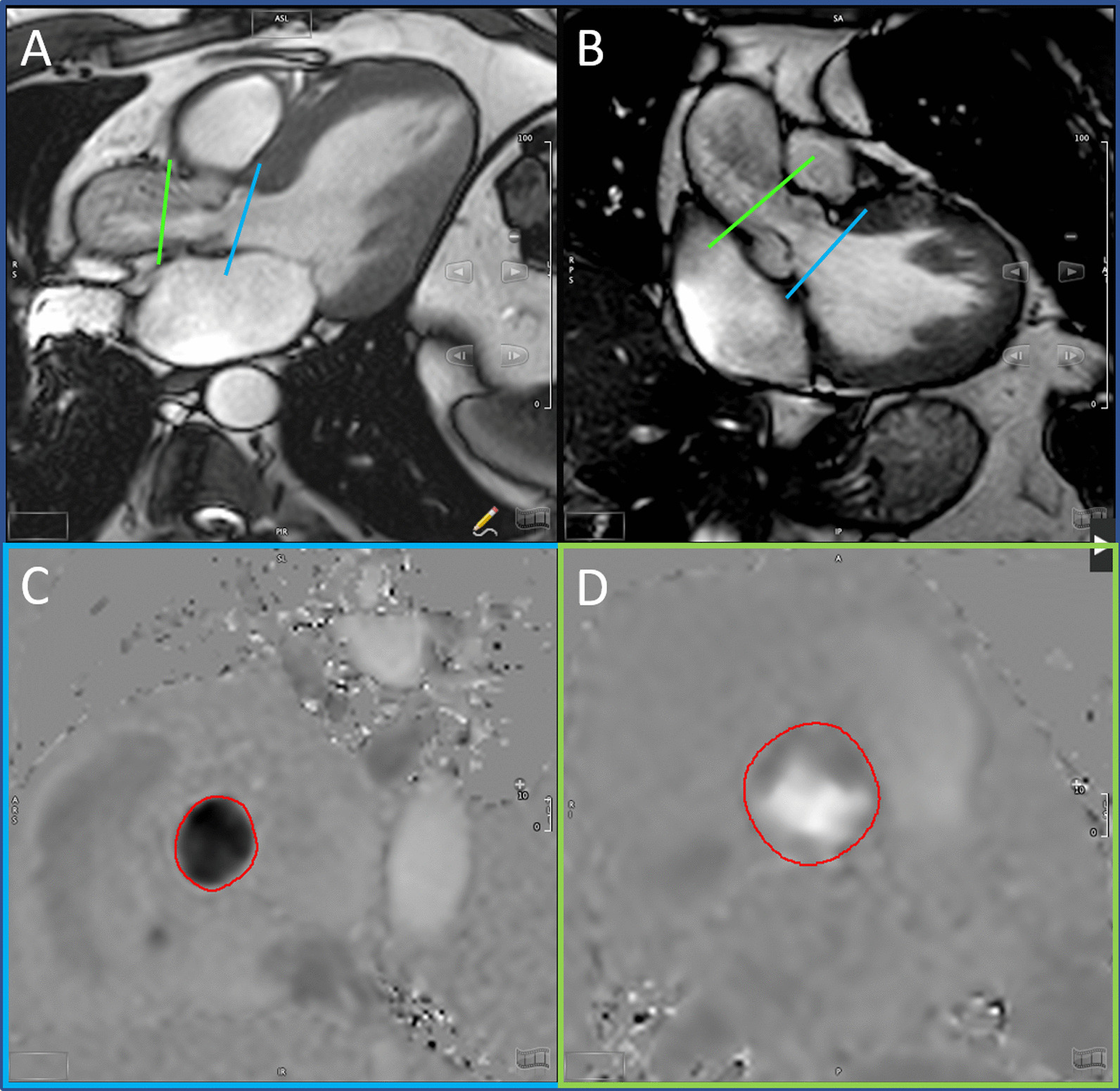
Locations of Phase-contrast flow measurements. **A**, **B** show double oblique orthogonal planes of the left ventricular (LV) outflow tract (LVOT) with the corresponding slice planes for the LVOT (blue lines) and ascending aorta (green lines). Phase-contrast images for measurement of flow at the LVOT and ascending aorta are shown in **C**, **D**

**Fig. 2:**
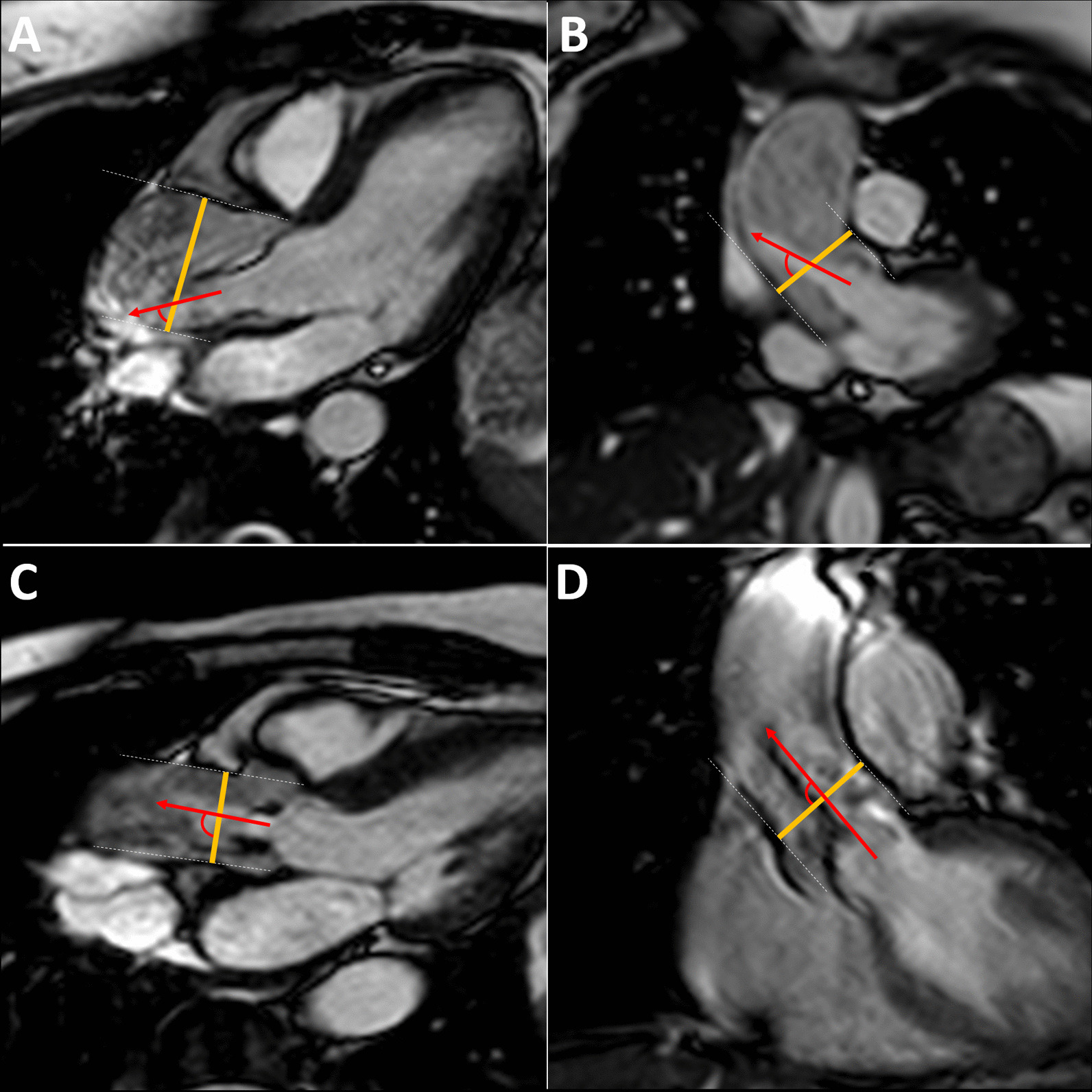
Assessment of jet eccentricity. **A**, **B** double-oblique long-axis of a bicuspid aortic valve with an eccentric jet (jet angle 65 degrees). **C**, **D** trileaflet aortic valve with a centrally aligned jet (jet angle 89 degrees). Orange lines: Site of phase contrast planes; Red arrows: direction of flow

Using double-oblique long-axis views of the LVOT and aortic valve, through-plane PC imaging was performed during breath-hold at two sites: (1) LVOT, 5–10 mm below the aortic annulus in mid-systole and (2) AAo, 10-mm above the aortic annulus (Fig. 1). The imaging PC sequence was planned parallel to the aortic valve annulus plane as previously described [16]. Flow imaging parameters consisted of TR/TE = 4.29–4.92/2.52–3.05 ms, flip angle = 15°, 24 phases, pixel spacing = 1.32–2.07 mm, slice thickness = 10 mm, acquisition matrix = 256 × 208. For each patient, peak aortic jet velocity measured by TTE was used as a starting point to define CMR encoding velocity in the AAo [CMR encoding velocity = (1.25–1.5) × peak jet velocity] with further adjustment in case of aliasing. Forward systolic flows (SV_AAo_ and SV_LVOT_) were computed using semi-automated tracings. Regurgitant volume was measured by PC at both sites and also estimated using the difference between right ventricular (RV) and LV SVs as these patients did not have significant mitral or tricuspid regurgitation [15]. We have evaluated the eccentricity of the aortic stenosis jet by assessing the angle between the aortic valve plane and the aortic jet in double-oblique long axis images. An angle of 90° reflects a jet flow parallel to the vessel orientation, and lower angles reflects jet eccentricity (Fig. 2). The angle was measured in 2 cross sectional planes, and the lowest measured angle was registered. Mathematical correction of the measured SV_AAo_ for the eccentricity angle was performed [corrected flow = measured flow/sin(angle)] as illustrated in Additional file 1: Figure S1). All analyses were done with cvi42 software (version 5.6.4, Circle Cardiovascular Imaging, Calgary, Alberta, Canada).

### Statistical analyses

Normal distribution of continuous variables was assessed using the Shapiro–Wilk test. Continuous data were expressed as mean ± standard deviation or median [interquartile range], and categorical variables as percentages. Correlation and agreement (95% confidence intervals) between SV_AAo_ and SV_LVOT_ as compared with SV_VM_ were assessed by Spearman correlations and Bland–Altman comparisons [17]. Paired Student *t* tests were used to test for significance of any overestimation or underestimation. A margin of error of ± 10% between PC derived SV and SV_VM_ was considered concordant measurements, and any difference exceeding this limit was considered a significant underestimation or overestimation. Receiver operating curves (ROC) were performed to derive the best thresholds for each parameter associated with discordance. Variability of measurements for SV_LVOT_ and SV_AA_ vs SV_VM_ were stratified according to aortic stenosis severity, aortic valve morphology and eccentricity of the jet. Statistical analyses were performed with STATA (version 15.3, Stata Corporation, College Station, Texas, USA). A two-sided *p* value < 0.05 was considered significant.

## Results

### Study population

Aortic stenosis severity ranged from none to severe [peak velocity 2.3 (1.7–3.0) m/s, mean gradient 13 (4, 7–19) mmHg]. Demographic, echocardiographic and CMR characteristics are depicted in Table 1. A bicuspid aortic valve was present in 36 (41%) patients. No patient had more than trace mitral regurgitation. Aortic diameter was higher in bicuspid vs tricuspid patients (3.6 ± 0.5 vs 3.4 ± 0.4 cm respectively, p = 0.037). Thirty-three (38%) patients were scanned at 1.5T and 55 (62%) at 3T.

**Table 1:** Baseline characteristics

Clinical data	All patients (n = 88)
Age, years	55 [31–69]
Male sex, n (%)	60 (68%)
Bicuspid aortic valve, n (%)	36 (41%)
Echocardiographic data	
Peak aortic valve velocity, m/s*	2.3 [1.7–3.0]
Mean gradient*	13 [4, 7–19]
Aortic stenosis severity*	
Aortic sclerosis (Vmax < 200 cm/s)	31 (35%)
Mild (Vmax 200–300 cm/s)	38 (43%)
Moderate (Vmax 300–400 cm/s)	13 (15%)
Severe (Vmax > 400 cm/s)	6 (7%)
Aortic regurgitation*	
None/trace	66 (75)
Mild	16 (18)
Moderate	6 (7)
Cardiovascular magnetic resonance data	
Eccentric jet (angle < 85°), n (%)	45 (51%)
Among trileaflet valve	19 (36%)
Among bicuspid valve	26 (72%)
LVEDV, ml	155 ± 40
LVEDV (excluding PM), ml	138 ± 35
LVESV, ml	67 ± 24
LVESV (excluding PM), ml	52 ± 19
LVEF, %	57 ± 6
LVEF (excluding PM), %	63 ± 7
RVEDV, ml	159 ± 41
RVESV, ml	74 ± 25
RVEF, %	54 ± 6
Aortic diameter, cm	3.5 ± 0.4
Among trileaflet valve	3.4 ± 0.4
Among bicuspid valve	3.6 ± 0.5
Stroke volume estimations	
LV stroke volume (volumetric), ml	87 ± 20
LV stroke volume (volumetric, excluding PM), ml	86 ± 20
LV stroke volume (PC_AA_), ml	80 ± 20
LV stroke volume (PC_LVOT_), ml	84 ± 20
RV stroke volume (volumetric), ml	85 ± 19

*By Echocardiography. Data presented as count (%), mean ± standard deviation or median [interquartile range] according to variable distribution. *LV* left ventricular, *RV* right ventricular, *EDV* end-diastolic volume, *ESV* end-systolic volume, *EF* ejection fraction, *PC* phase-contrast, *PM* papillary muscles, *SV* stroke volume

### Forward stroke volume estimation according to different methods

**Fig. 3:**
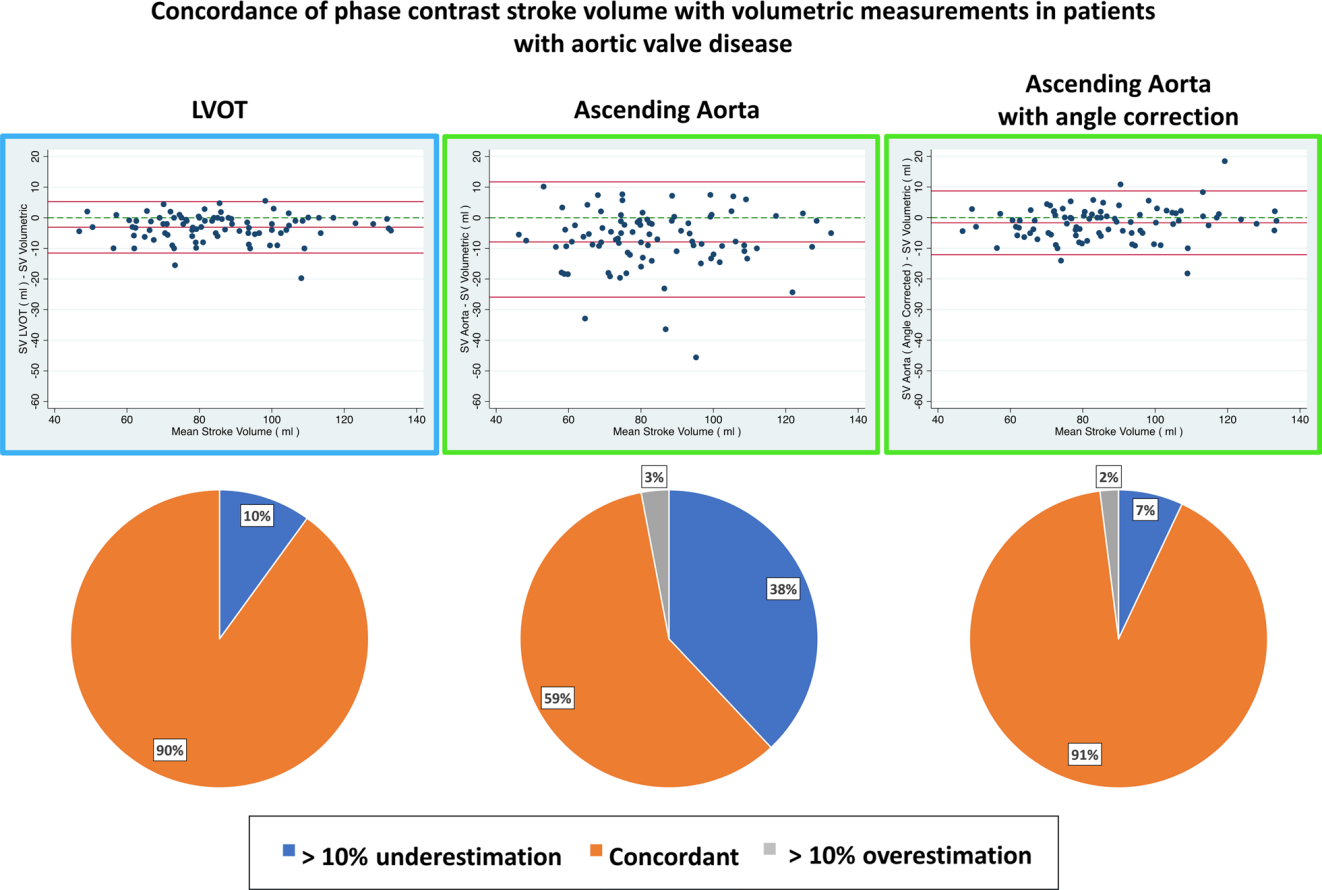
Agreement between PC_AA_, PC_LVOT_ and volumetric method. Upper Panels: Bland–Altman plots comparing stroke volume (SV) estimated by phase contrast (PC) at the LVOT, AAo and corrected AAo flow respectively as compared to the reference (volumetric method). Data presented included the papillary muscles in the blood pool (similar results obtained by excluding them). Solid red lines: mean bias ± 2 standard deviations. Dashed green line: level of zero bias. Pie charts show the proportion of concordance, over- and under-estimation of SV for 3 methods

LV SVs by different methods are shown in Table 1. Overall, correlation between SV_AAo_ and SV_LVOT_ was excellent (r = 0.89, p < 0.001, Additional file 1: Figure S2). However, SV_AAo_ lead to lower SV values than SV_LVOT_, while SV_VM_ was statistically higher than both SV_LVOT_ and SV_AAo_ (both p < 0.001, Additional file 1: Table S1). Exclusion of the papillary muscles from the blood pool led to significantly lower end-diastolic and end-systolic volumes and significantly higher LVEF (Table 1). SV, albeit with a statistically significant difference (86 ± 20 ml vs 87 ± 20 ml excluding and including papillary muscles within LV mass respectively), was clinically comparable (average difference 1 ± 5 ml). SV_LVOT_ was concordant with SV_VM_ in 90% of the cases, vs 59% for SV_AAo_ (p < 0.001, Fig. 3). Similar results were obtained when papillary muscles were excluded from blood pool (93% vs 59% concordance for SV_LVOT_ vs SV_AAo_ respectively, p < 0.001). CMR field strength had no impact on the discordance between SV_AAo_ and SV_LVOT_ compared to SV_VM_ (p = 0.12). The use of background static tissue correction in discordant cases did change the SV by an average of 1 ± 1 ml in the LVOT and 1 ± 1 ml in the aorta, without changing the concordant/discordant status in any case.

### Factors associated with discordance between SV_AAo_ and SV_VM_

**Fig. 4:**
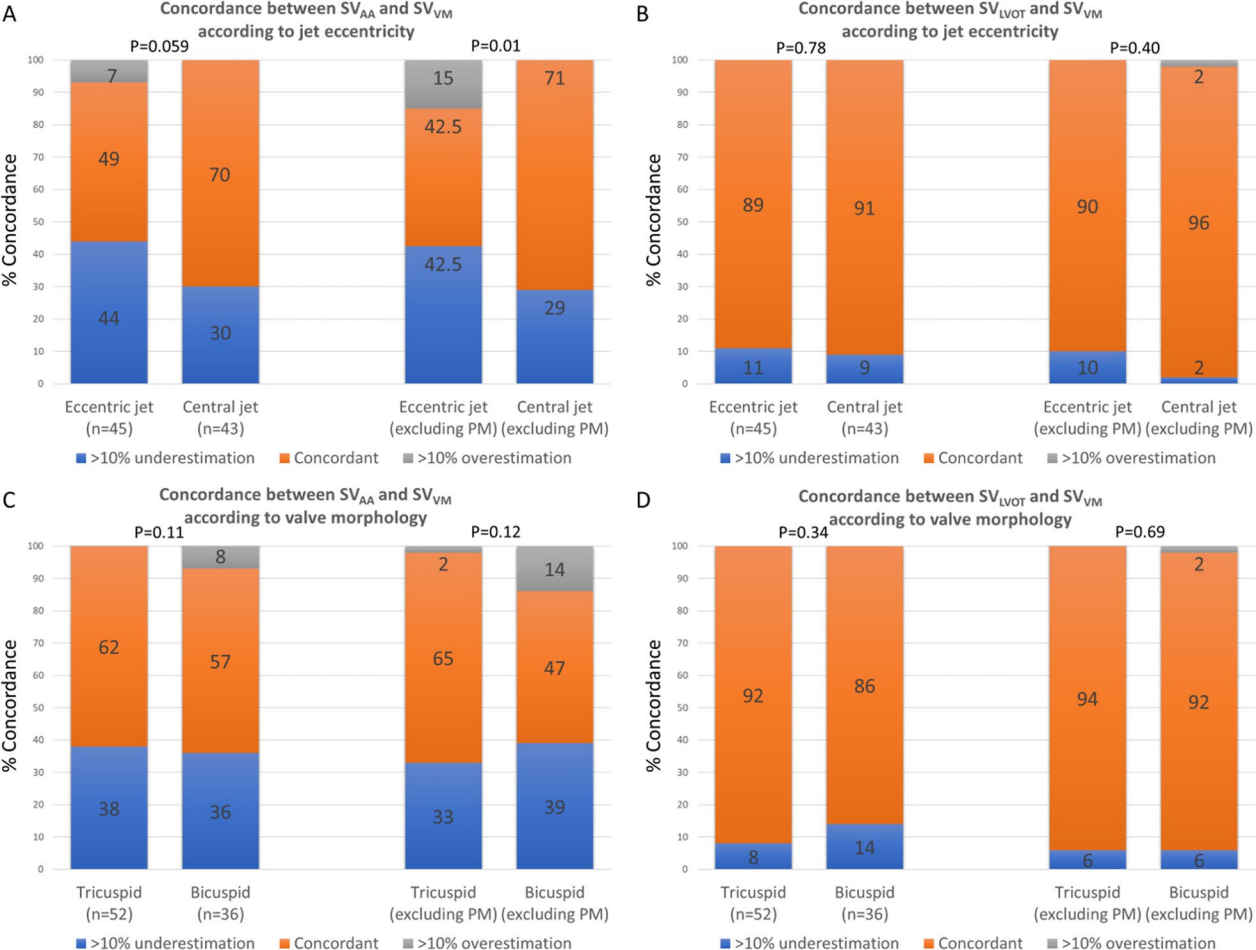
Concordance according to jet eccentricity and valve morphology. Bar charts representing the proportion of concordance, under and overestimation according to jet eccentricity (**A**, **B**) and valve morphology (**C**, **D**) for SV_AAo_ (left panels) and SV_LVOT_ (right panels)

There was no relationship between the degree of underestimation of SV estimated by SV_AAo_ as compared to SV_VM_ and peak aortic velocity (r = − 0.14, p = 0.19). Difference between SV_AAo_ and SV_VM_ was related to the jet angle (more discordance in more eccentric jets, Additional file 1: Figure S3). ROC analysis suggested an angle of 85 degrees as the best threshold to predict SV_AAo_ vs SV_VM_ discordance (Additional file 1: Figure S4)_._ A jet angle < 85º was present in 45 (51%) patients and was more frequent as aortic stenosis severity increases [aortic sclerosis: 3 (10%); mild aortic stenosis: 22 (58%); moderate aortic stenosis 10 (77%); severe aortic stenosis: 5 (83%), p < 0.001]. In central jets (angle 85–90°), bias between SV_AAo_ and SV_VM_ was lower than for eccentric jets (absolute difference 6 ± 6 ml vs 12 ± 9 ml respectively, p < 0.001). Concordance of SV_AAo_ was significantly higher in central vs eccentric jets (Fig. 4). Mathematical correction for the eccentricity angle however restored the concordance with SV_VM_ (91% concordance after correction) with lower overall bias (Table 1).

There was a non-significant trend for better concordance in patients with trileaflet vs bicuspid valves (Fig. 4). Patients with a bicuspid valve had however more frequently eccentric jets than those with trileaflet morphology (65% vs 35%, p < 0.001) and had higher peak aortic velocities [2.7 (2.4–3.0) m/s vs 2.2 (1.9–2.4 m/s), p < 0.01]. After multivariate adjustment for valve morphology, eccentricity and peak aortic velocity, the only variable that remained associated with discordance between SV_AAo_ and SV_VM_ was eccentricity of the jet, either as a continuous variable (jet angle) or dichotomized as central jet/eccentric jet (Table 2). There was no association between the aortic diameter and the degree of discordance between SV_AAo_ and SV_VM_ (p = 0.26).

**Table 2:** Univariate and multivariate analyses of correlates with absolute discordance between PC_AAo_ and SV_VM_

	Univariate		Multivariate			
			Model 1		Model 2	
	Standardized β coefficient ± SE	P value	Standardized β coefficient ± SE	P value	Standardized β coefficient ± SE	P value
Peak aortic velocity	0.17 ± 0.01	0.11	0.04 ± 0.01	0.72	0.05 ± 0.01	0.69
Bicuspid valve	0.18 ± 1.71	0.10	0.05 ± 1.79	0.63	0.04 ± 1.88	0.76
Eccentric jet	0.35 ± 1.60	0.001	0.32 ± 1.80	0.007		
Jet angle (°)	− 0.32 ± 0.11	0.003			− 0.28 ± 0.13	0.02

*PC* phase contrast, *SE* standard error

### Factors associated with discordance between SV_LVOT_ and SV_VM_

There was no significant predictor of SV_LVOT_/SV_VM_ discordance for the studied variables (peak aortic velocity: p = 0.22, jet angle: p = 0.21, aortic diameter: p = 0.58, valve morphology: p = 0.54).

### Assessment of aortic regurgitation

The grade of aortic regurgitation as determined by echocardiography was none/trace in 66 (75%) patients, mild in 16 (18%) and moderate in 6 (7%) patients. No patients had severe aortic regurgitation as per exclusion criteria. Regurgitant volume by PC in the LVOT was 30% smaller vs the values obtained in the aorta in the whole cohort (3 ± 3 ml vs 5 ± 4 ml, p < 0.01) and a similar numerical trend was observed in the 6 patients with moderate AR (10 ± 8 vs 14 ± 7 ml, p = 0.25). Regurgitant volume estimated in the AAo correlated better with the difference between RV and LV SVs (Additional file 1: Figure S5).

## Discussion

The main findings of this study are: (1) in patients with aortic stenosis, SV_LVOT_ has better overall agreement to volumetric measurements than SV_AAo_; (2) jet eccentricity is the main factor associated with discordant SV_AAo_; (3) mathematical correction using measured SV and eccentricity angle corrected the discordance in our population and (4) consistent with previous studies and current recommendation, aortic regurgitant volume is likely underestimated when assessed in the LVOT. To the best of our knowledge this is the first study to formally explore the validity of PC CMR measurement site in a population of this size with various degrees of aortic stenosis severity.

### Aortic stenosis, turbulent jets and phase-contrast CMR

PC-CMR is a powerful, accurate and reproducible non-invasive tool to assess blood flow [1, 18]. However, some caveats should be considered: (1) Acquisition plane should be reasonably perpendicular to the direction of flow; this direction is not always in line with the anatomic orientation of the cavity/vessel in which the flow is measured. In some cases, 2 or more jets differentially oriented may co-exist, and can also change their direction throughout the cardiac cycle. Also, high velocity jets may provoke signal loss due to flow acceleration and intravoxel dephasing [10]. Therefore, aortic stenosis is challenging as it presents both problems: high velocity jets which are frequently eccentric. Use of a plane upstream of the stenotic lesion (LVOT) might circumvent these problems.

Interestingly, there was no relation between SV_AAo_ discordance and peak aortic velocity in our cohort. This suggest that aortic stenosis hemodynamic severity might not be by itself a reason to use another site to measure SV. Importantly, the most severe spectrum of aortic stenosis is underrepresented in the current cohort, and this absence of relation could be related to low statistical power. Also, PC-CMR imaging has made advances since its first implementation, with shorter echo-times minimizing the impact of accelerating flow. SV_AAo_ was acquired at a plane in the ascending aorta approximately 10 mm from the valve, which is distal to the vena contracta [16]. Previous works showed that shorter echo-times and distance from the stenosis can reduce the error in PC assessment of flow [6, 10, 16]. While we did not acquire flow data more distally in the AAo, it is likely that the discordance would decrease as the measurement site moves away from the stenosis.

The only factor independently associated with the degree of discordance between SV_AAo_ and SV_VM_ in our cohort was jet eccentricity. It is known that stroke volume measurement requires an imaging plane positioned orthogonal to the main direction of flow [1, 3, 18]. However, in cases of eccentric jets (which are misaligned to the main longitudinal axis of the aorta), optimal PC planning can be extremely challenging and time-consuming. Measurement of SV at the LVOT showed improved accuracy compared to the AAo. Interestingly, mathematical correction for the eccentricity angle improved the concordance which became similar to what is observed with the LVOT measures. Regarding valve morphology, previous studies have shown that in bicuspid patients, flow measurement at the AAo lead to underestimation of forward flow [19]. However, bicuspid valves are frequently associated with complex flow patterns. Our results suggest that bicuspid valve morphology is more often associated with eccentric jets, but otherwise not directly associated with the degree of discordance.

### Clinical relevance for CMR and aortic regurgitation quantification.

CMR is increasingly suggested to assess the severity of mitral regurgitation and aortic regurgitation. Several studies have evaluated the reliability of mitral regurgitation quantification by CMR, including systematic review of more than 30 studies [20–22]. The PC plane used to derive aortic forward flow was mostly the AAo at sinotubular junction. Most studies do not mention the use of SV_LVOT_ or another site as an alternative to SV_AAo_, and did not include patients with concomitant aortic stenosis; however coexisting aortic and mitral diseases are frequent in real life practice. It is estimated that up to 20% of patients have at least two moderate valvular pathologies and this will likely expand in the future as the prevalence is constantly increasing [23, 24]. Regarding aortic regurgitation quantification, potential limitations of PC have been discussed in the presence of non-laminar flow [25]. Our study was not designed to assess the best PC plane for aortic regurgitation and is limited by a small number of patients with significant aortic regurgitation. Nevertheless, our results show 30% difference between LVOT and AAo, while the AAo correlated better with RV-LV SV differences. This is consistent with previous studies [11] and suggest that in the case of mixed aortic disease with both stenosis and regurgitation, using 2 sites (LVOT for forward flow; AAo for regurgitant flow) might provide the best assessment.

Despite its good performance, the use of LVOT can be limited in case of subvalvular flow acceleration (hypertrophic cardiomyopathy/sub-aortic membrane). In this case, the selection of an alternative site to confirm forward SV is advisable. The use of either an aortic plane as distal as possible from the flow turbulence, right sided PC planes or combination of both can be considered. Suggested approaches for PC planning in different clinical scenarios are presented in Table 3.

**Table 3:** Phase contrast suggested plane locations in patients with valvular disease, for which left heart stroke volume is needed to compute mitral regurgitation or shunt

Aortic valve status	Best plane for phase contrast forward stroke volume	Comments
No evidence of aortic disease	Ascending aorta	Ascending aorta is the easiest plane to acquire Mitral regurgitation volume = SV_VM_ − systolic SV_AAo_
Presence of aortic stenosis without aortic regurgitation	Left ventricular outflow tract Alternatives: 1. Ascending aorta: if the flow is aligned with the vessel. Consider angle correction in case of eccentric jets 2. Right-sided phase contrast is reasonable in absence of shunt or aortic/pulmonary regurgitation	Mitral regurgitation volume = SV_VM_ − systolic SV_LVOT_ Limitation: aortic regurgitation cannot be assessed accurately using the LVOT flow If SV_AAo_ is used, a plane in the distal ascending aorta is preferable to minimize the effect of turbulent flow
Presence of aortic regurgitation or mixed aortic disease Need for aortic regurgitation quantification	Left ventricular outflow tract AND Ascending aorta Alternative: Single plane in the ascending aorta if the flow is aligned with the vessel. Consider angle correction in case of eccentric jets	Mitral regurgitation volume = SV_VM_ − systolic SV_LVOT_ Forward aortic SV: systolic SV_LVOT_ Aortic regurgitation volume: diastolic flow in the AAo If SV_AAo_ is used, a plane in the distal ascending aorta is preferable to minimize the effect of turbulent flow
Presence of LVOT obstruction (mitral systolic anterior motion, septal hypertrophy, sub-aortic membrane)	Ascending aorta, especially for aortic regurgitation volume Can be combined to right-sided phase contrast to confirm determine total effective SV in the absence of shunt LVOT is likely not valid for either forward or regurgitant flow	Aortic regurgitation volume: diastolic flow in the AAo Mitral regurgitation volume: SV_VM_—SV_right side_—AR volume (if no shunt); or SV_VM_—systolic SV_AAo_ (consider angle correction if eccentric jet) If systolic SV_AAo_ is used, a plane in the distal ascending aorta is preferable to minimize the effect of turbulent flow Potential limitation should be acknowledged when using multiple PC planes as the risk of error increases with the number of measurements

### Limitations

Our data are from a single center study, and the population limited to the inclusion criteria of the PROGRESSA study with a low prevalence of severe aortic stenosis. We cannot exclude that very high maximal velocity aortic stenosis can influence SV measurement in the aorta, even in central jets. AAo dilatation is a potential cause of flow turbulence; while the association with discordance was not significant, we had few patients with dilated AAo. Also, few patients had significant aortic regurgitation because of the specific nature of the cohort study which includes mainly patients with aortic stenosis. There was no phantom correction for the flow acquisitions. The aortic phase contrast sequences were planned relatively close to the valve (10 mm): a plane closer to the aortic arch was not assessed but could have decreased discordance. The mathematical correction used in this study does not take into account the whole complexity of flow turbulence and will require validation in other cohorts. PC planning in the LVOT can be potentially challenging, however imaging quality was excellent in all cases with good concordance with SV_VM_. The assessment of other sites (right sided PC, combination of descending aorta/superior vena cava) has not been explored. Coronary flow can explain in part the difference between SV measured in AA vs LVOT—however as coronary perfusion is in diastole and we have measured systolic forward flow, this component has likely a minor impact. Finally, time-resolved 3D PC (4D-flow) was not performed. 4D flow has been shown to overcome some of the 2D PC-CMR limitations and is an extremely promising tool in this field [26].

## Conclusion

Aortic stenosis can negatively influence the PC SV_AAo_. SV_LVOT_ has overall better agreement with SV_VM_ than SV_AAo_, especially in patients with eccentric jets. Therefore, flow jet direction rather than aortic stenosis severity alone should be assessed to select the best plane for SV measurement. However, LVOT plane underperforms for aortic regurgitation quantification. Thus, the use of an additional PC-CMR plane at the LVOT in addition to -but not instead of- the conventional plane at the AAo might be preferable in patients with mixed aortic disease. Mathematical correction of SV_AAo_ for eccentric jets should be explored in future studies.

## Supplementary Information

**Additional file 1.** Supplementary material.

## Data Availability

The datasets generated and/or analysed during the current study are not publicly available due to the ongoing status of the main clinical study, but are available from the corresponding author on reasonable request.

## Abbreviations

AAo: Ascending aorta
CMR: Cardiovascular magnetic resonance
LV: Left ventricle/left ventricular
LVEDV: Left ventricular end-diastolic volume
LVEF: Left ventricular ejection fraction
LVESV: Left ventricular end-systolic volume
LVOT: Left ventricular outflow tract
PC: Phase-contrast
RV: Right ventricle/right ventricular
RVEDV: Right ventricular end-diastolic volume
RVEF: Right ventricular ejection fraction
RVESV: Right ventricular end-systolic volume
SV: Stroke volume
SV_AAo_: Stroke volume measured at the ascending aorta (phase-contrast)
SV_LVOT_: Stroke volume measured at the LVOT (phase-contrast)
SV_VM_: Stroke volume measured using volumetric method
TTE: Transthoracic echocardiography
