# Supplementary material for: Accuracy of stroke volume measurement with phase-contrast cardiovascular magnetic resonance in patients with aortic stenosis
Source: J Cardiovasc Magn Reson. 2021 Nov 4;23:124. doi: 10.1186/s12968-021-00814-4 (PMC8567621; doi:10.1186/s12968-021-00814-4)
Supplement: Supplementary file 1 — Additional file 1. Supplementary material. [file 12968_2021_814_MOESM1_ESM.docx]

**Accuracy of Stroke Volume Measurement with Phase-Contrast Magnetic Resonance in Patients with Aortic Stenosis**

**SUPPLEMENTARY MATERIAL**

**Figure S1: Mathematical correction for eccentric flow.
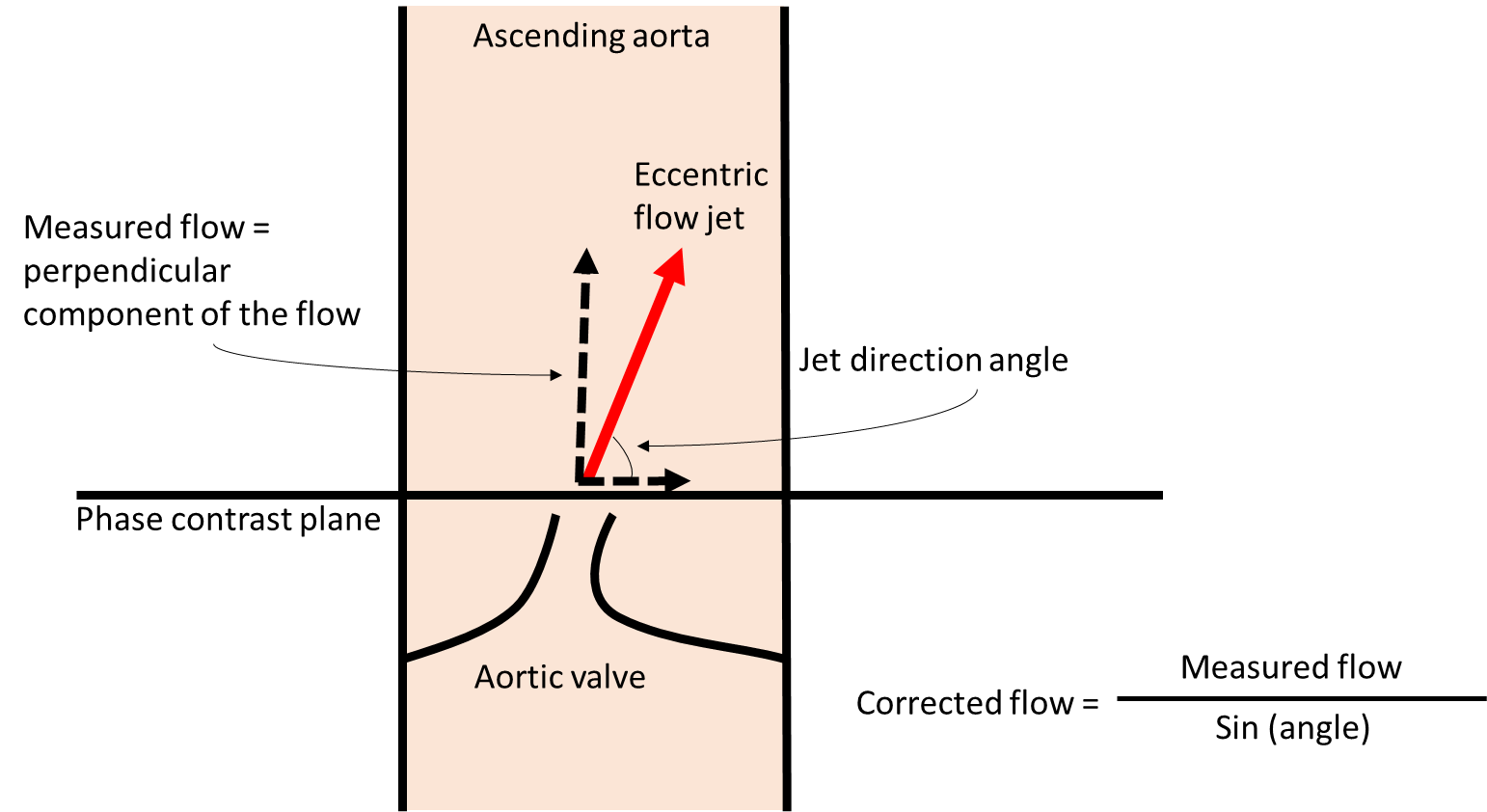
**

An estimation of the real flow was estimated using a trigonometrical function incorporating the measured flow with the measured eccentricity angle.

**Supplementary Table 1.** Overall bias between different methods

| Comparison | Bias (ml) | P value |
| --- | --- | --- |
| SV_AA_ - SV_LVOT_ | -4.0±8.3 | <0.001 |
| SV_AA_ - SV_VM_ | -7.1±9.6 | <0.001 |
| SV_LVOT_ - SV_VM_ | -3.1±4.3 | <0.001 |
| SV_RV_ – SV_VM_ | -2.5±5.8 | <0.001 |
| SV_VM_ – SV_VM (excluding PM)_ | 1.4±5.2 | 0.01 |
| SV_AA_ – SV_VM (excluding PM)_ | -5.7±9.5 | <0.001 |
| SV_LVOT_ – SV_VM (excluding PM)_ | -1.7±4.9 | 0.0017 |
| SV_AA (angle corrected)_ - SV_LVOT_ | +1.4±2.7 | <0.001 |
| SV_AA (angle corrected)_ - SV_VM_ | -1.7±5.3 | 0.004 |
| SV_AA (angle corrected)_ – SV_VM (excluding PM)_ | -0.3±5.4 | 0.63 |

*SV_AA_: stroke volume at the ascending aorta; SV_LVOT_: stroke volume at the LVOT; SV_VM_: left ventricular stroke volume (volumetric method); SV_RV_: right ventricular stroke volume (volumetric method)*

******Figure S2. Agreement and Correlation between SV_AA_ and SV_LVOT_**

R=0.89

P<0.001

Agreement (left panel) and correlation (right panel) between **SV_AA_ and SV_LVOT._** Left panel: The solid red lines represent the mean bias and ± 2 standard deviations. The dashed green line is the level of zero bias. Right panel: the solid red line represents the linear fit.

**Figure S3. Discordance between SV_AA_ and SV_VM_ according to jet angle**

Agreement (left panels) and correlation (right panels) between **SV_AA_ and SV_VM_** plotted against jet angle (higher jet angle meaning more centrally oriented jets) including (upper panels) and excluding (lower panels) papillary muscles.

Left panels: The solid red lines represent the mean bias and ± 2 standard deviations. The dashed green line is the level of zero bias. Right panel: the solid red line represents the linear fit.

**Figure S4. Receiver Operating Characteristics Curve for Concordance according to Jet Angle**

Receiver Operating Characteristics Curve for Jet Angle and Concordance between stroke volumes obtained by volumetric method and phase contrast in the aorta. The best threshold to predict discordance >10% between both methods was an angle of 85 degrees.

**Figure S5. Receiver Operating Characteristics for aortic regurgitation**

**
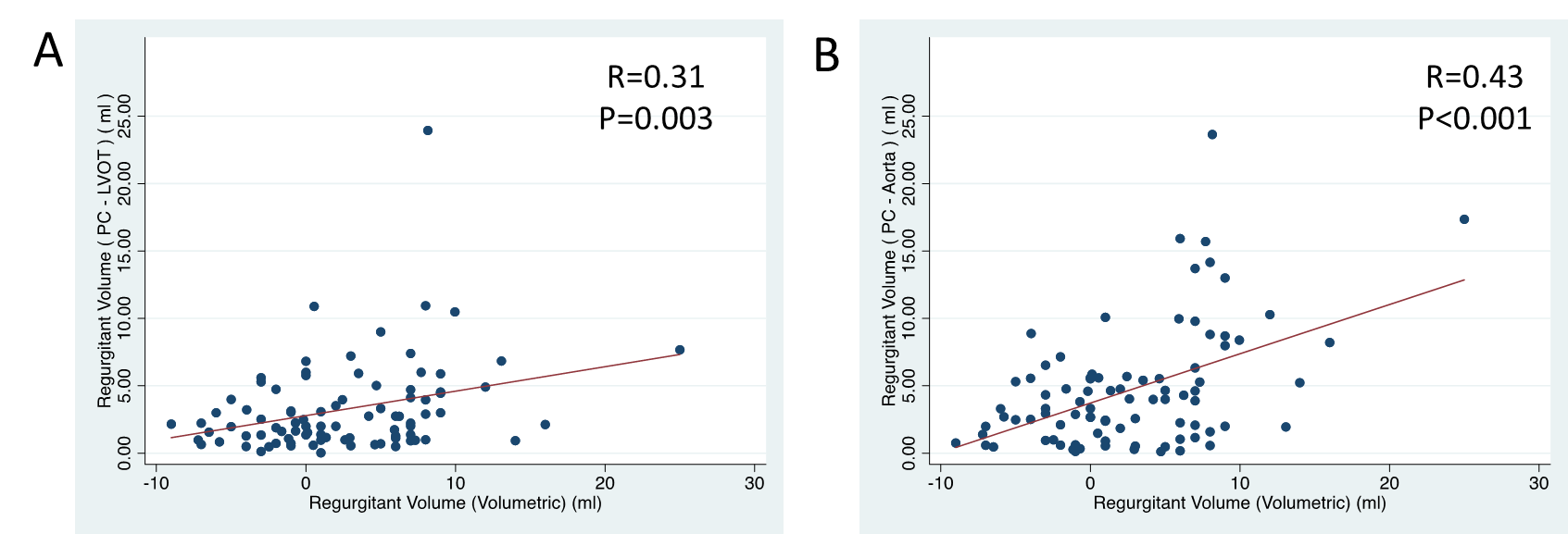
**

Correlation of phase contrast regurgitant volume (A: LVOT; B: ascending aorta) with right/left stroke volume difference as a surrogate for aortic regurgitation volume.
